# Report of metastatic ileal neuroendocrine tumor to the submandibular gland

**DOI:** 10.1016/j.ijscr.2018.10.028

**Published:** 2018-10-19

**Authors:** David Forner, Peter Cho, Martin Bullock, Daniel Rayson, S. Mark Taylor, Robert D. Hart, Jonathan R. Trites, Matthew H. Rigby

**Affiliations:** a Division of Otolaryngology – Head & Neck Surgery, Department of Surgery, Dalhousie University, Halifax, Nova Scotia, Canada; b Faculty of Medicine, Dalhousie University, Halifax, Nova Scotia, Canada; c Department of Pathology, Dalhousie University, Halifax, Nova Scotia, Canada; d Division of Medical Oncology, Department of Medicine, Dalhousie University, Halifax, Nova Scotia, Canada; e Present affiliation: Division of Otolaryngology – Head & Neck Surgery, Department of Medicine, University of Calgary, Alberta, Canada

**Keywords:** FNA, Fine needle aspiration, HPF, High powered field, MIBG, Metaiodobenzylguanidine, NET, Neuroendocrine tumor, SSA, Somatostatin analogue, Neuroendocrine tumor xx, Carcinoid syndrome, Submandibular gland, Metastasis

## Abstract

**Background:**

Neuroendocrine tumors (NETs) of small intestinal origin are generally slow-growing tumors with a relatively high propensity for metastases to surrounding organs and lymphatic tissue. We present the first case of an ileal NET metastasizing to the submandibular gland in a woman with metastatic carcinoid syndrome.

**Case presentation:**

A 55-year-old female presented with a four-month history of a palpable, left-sided neck mass. The patient had a history of metastatic neuroendocrine tumor of ileal origin, initially treated with primary resection 4.5 years previously, with known subdiaphragmatic metastases to the liver, mesenteric nodes, and peritoneum. Four years following primary resection she developed carcinoid syndrome leading to therapy with radiolabelled metaiodobenzylguanidine (MIBG), as well as telotristat etiprate in the context of a clinical trial due to progressive symptoms. A fine needle aspiration biopsy of the neck mass revealed an immunohistochemical staining pattern consistent with ileal NET. The patient underwent a left level 1b neck dissection and submandibular gland excision. Pathology was consistent with metastastic ileal NET.

**Conclusion:**

We report the first case of ileal NET metastasis to the submandibular gland. Familiarity with the carcinoid syndrome and associated physiology should be maintained as it can affect the head and neck on rare occasions. Maintaining a broad differential is key in diagnosis of undifferentiated neck masses.

## Highlights

• The differential diagnosis for neck masses is broad and includes neoplastic, infectious, and anatomic considerations.
• Bronchopulmonary neuroendocrine tumors are known to metastasize to the head and neck.
• This report is the first of ileal neuroendocrine tumor metastasis to the submandibular gland.

## 1 Introduction

Neuroendocrine tumors (NETs) have multifocal sites of origin and are relatively indolent in the majority of cases. They are the most common malignancies of the small intestine, with most originating in the ileum, and can give rise to carcinoid syndrome if metastases to the liver develop. The classic carcinoid syndrome is due to circulating serotonin secreted by metastatic, functional small intestinal NETs and results in progressive facial flushing, diarrhea, and wheezing [1].

Rare cases of metastasis from bronchopulmonary NETs to the head and neck have been reported, including the parotid glands [2] and thyroid [3]. We present the first case of an ileal NET metastasizing to the submandibular gland in a woman with metastatic disease to the liver and carcinoid syndrome. The case is reported in line with the SCARE criteria [4].

## 2 Case presentation

A 55-year-old female presented to the Otolaryngology – Head & Neck Surgery clinic with a four-month history of a left-sided neck mass. The patient had a history of metastatic ileal NET with metastases to the liver, mesenteric nodes, and peritoneum. She underwent primary right hemicolectomy and small bowel resection in 2010. Pathology revealed two foci of well-differentiated malignant NET in the terminal ileum, with muscle and serosal invasion, and three of nine regional lymph nodes involved. There were two to three mitoses per ten high power fields (HPFs) with no necrosis and the Ki-67 index was 3%. Immunohistochemistry revealed positivity for synaptophysin and chromogranin. Pre-operative imaging revealed metastatic involvement of an aorto-caval node, thus the disease was staged as pT4(m)N1M1. Post-operatively, treatment began with long acting somatostatin analogue (SSA). Liver metastases were detected eleven months after her initial surgery. In 2014, she developed carcinoid syndrome leading to therapy with radiolabelled metaiodobenzylguanidine (MIBG). Subsequently she received telotristat etiprate due to progressive symptoms.

On presentation of the neck mass in 2015, she had no additional otorhinolaryngological symptoms. Examination of the neck revealed a smooth, mobile, painless, 2-cm mass located in the submandibular triangle. The remainder of the physical examination was unremarkable, including flexible nasopharyngoscopy which revealed no mucosal masses or lesions in the upper aerodigestive tract.

**Fig. 1:**
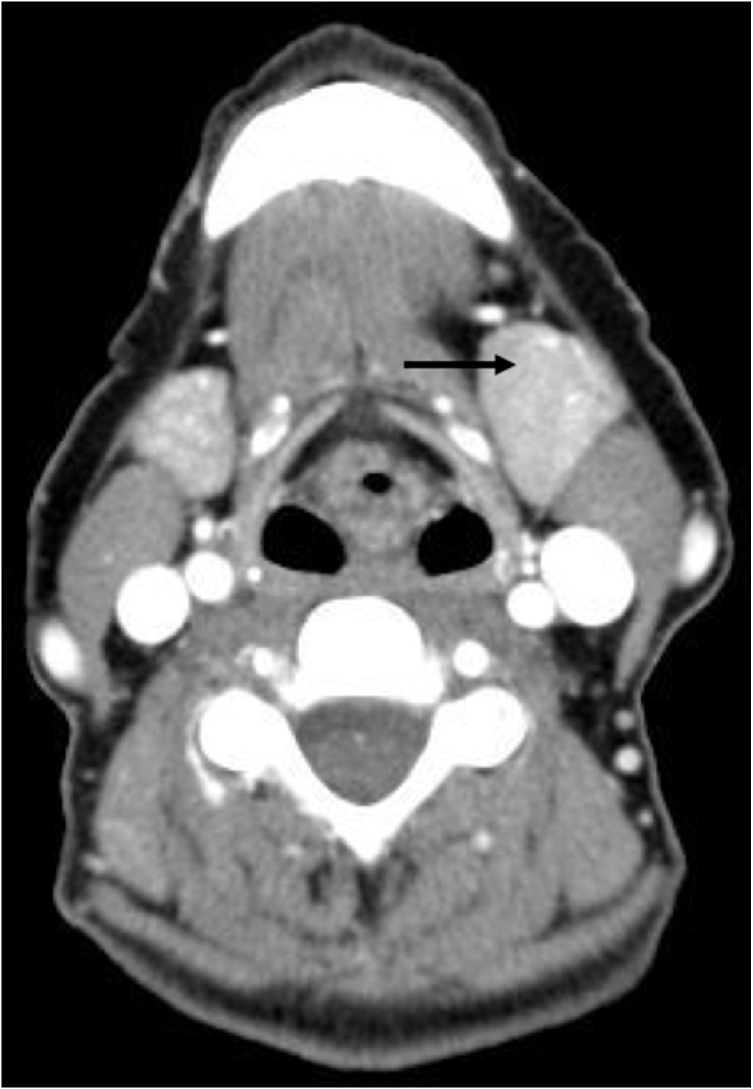
Computed tomography image of the patient’s neck demonstrates a homogeneous increase in the size of the left submandibular gland (arrow). No lymphadenopathy was present.

Fine needle aspiration (FNA) revealed malignant cells with mildly pleomorphic nuclei and salt and pepper chromatin. Immunohistochemistry was positive for chromogranin and synaptophysin, and consistent with metastatic NET. Computed tomography imaging of the mass revealed left submandibular gland enlargement and no cervical lymphadenopathy (Fig. 1). The multidisciplinary head and neck tumor board, in conjunction with her primary medical oncologist, recommended resection of the tumor to improve local control due to the low morbidity of the surgery.

**Fig. 2:**
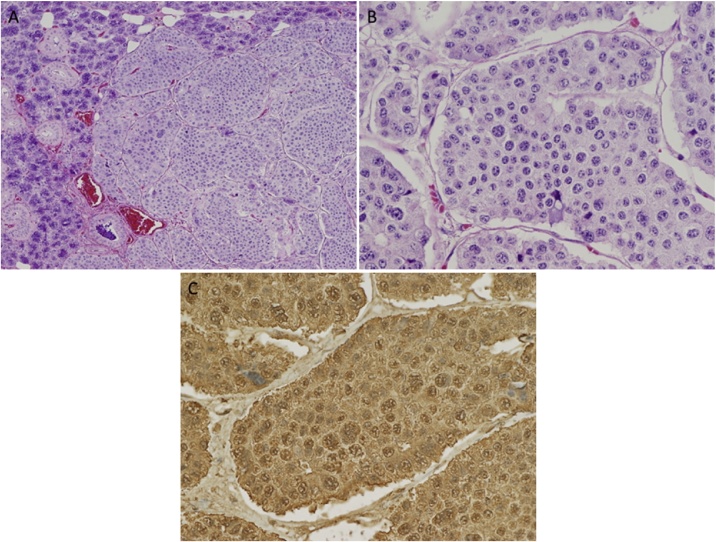
Permanent histopathology specimen of submandibular gland lesion (A) shows large regular islands of carcinoid tumor cells in a so-called organoid pattern on the right, with residual submandibular acini and ducts on the left (Hematoxylin & eosin staining; original magnification ×100). (B) On higher (×400) magnification, features characteristic of neuroendocrine tumor cells are apparent with hematoxylin & eosin staining showing cells with a characteristic coarsely granular “salt-and-pepper” chromatin. (C) Positive chromogranin immunostaining is consistent with neuroendocrine tumor cells.

An uncomplicated left level 1b neck dissection was performed and there were no permanent post-operative sequelae. Gross pathology revealed a circumscribed tumor mass within the submandibular gland that measured 2.2 × 1.5 × 1.4 cm, and appeared as a firm, smooth nodule with a grayish white color. The tumor displayed focal abutment of the margin, but did not extend outside of the gland. Immunohistochemistry was again positive for chromogranin and synaptophysin, consistent with the previous FNA and was (Fig. 2).

Unfortunately, the patient passed away one year later due to progressive disease in the mesentery and liver.

## 3 Discussion

Neuroendocrine tumors, although uncommon, are increasing in incidence. Data from the Surveillance, Epidemiology, and End Results (SEER) program found an approximate 5-fold increase in incidence between 1973–2004 (1.09–5.25 per 100,000), similar to data from Ontario [5,6]. They are characterized by generally indolent, but highly variable, growth patterns and the ability to secrete peptides leading to a constellation of systemic symptoms. The carcinoid syndrome consists of facial flushing and diarrhea, with a lesser incidence of bronchospasm, and arises in approximately 30% of patients with small intestinal NETs [7,8].

Multiple synchronous NETs are discovered in approximately 25% of patients with tumors of small intestinal origin, as was the case in our patient [6,8]. Current grading systems for NETs relies on cellular morphology and Ki-67 proliferation index. Well-differentiated disease is classified as grade 1 if Ki-67 index is ≤ 2% and grade 2 if between 3–20%. Tumors with Ki-67 index above 20% are classified as grade 3 with poorly-differentiated disease being classified as a neuroendocrine carcinoma (NEC) rather than a NET due to its more aggressive proliferative behavior [9].

Synchronous metastatic disease is present in approximately 20% of patients at diagnosis, with a further 35–40% developing metastatic disease at some point [5]. The most common sites of metastatic involvement for small intestinal NETs are the liver, mesentery, and peritoneum [6]. To our knowledge, metastasis to the salivary glands has not been described in NETs of gastrointestinal origin. Previous cases have reported bronchopulmonary primaries metastasizing to the submandibular [10], and thyroid glands [3], as well as metachronous metastasis to the parotid glands, thyroid, lip, and submandibular gland [11]. Intestinal NETs have been reported to metastasize to the orbits [12]. The presented case therefore represents the first case of an intestinal NET metastasizing to the submandibular gland. Of note, our patient developed this unusual metastatic focus approximately 4.5 years after her initial surgery and without any other new foci of disease in the interval between surgery and her death.

Therapeutic goals for NETs include curative-intent surgery when feasible, and tumor proliferation and symptom control in the setting of unresectable metastatic disease [13]. First-line therapy for the latter includes SSA therapy, which exerts both an anti-proliferative and anti-secretory effect. More recently, peptide radioreceptor therapy with Lutetium-177 and telotristat etiprate, has been approved for progression-free survival and symptom control in the setting of progressive disease despite SSA therapy [[14], [15], [16]].

## 4 Conclusion

NETs of small intestinal origin are slow-growing tumors with a relatively high propensity for metastases to surrounding organs and lymphatic tissue. We report the first case of an ileal NET metastasizing to the submandibular gland. For all clinicians, awareness of NETs and the carcinoid syndrome is important due to rising incidence and the fact that diagnosis is often delayed due to the symptom non-specificity or absence. For otolaryngologists, maintaining a broad differential for the initial presentation of a neck mass in the setting of an uncommon metastatic disease is also important. Expert pathology review and comparison with original tumor specimens is critical to minimize risk of misdiagnosis.

## Conflict of interest

The authors declare that they have no conflict of interest.

## Funding

This research did not receive any specific grant from funding agencies in the public, commercial, or not-for-profit sectors

## Ethical approval

No research ethics approval was necessary for this case report. Written informed consent was obtained from the patient for publication of this Case report and any accompanying images. A copy of the written consent is available for review by the Editor-in-Chief of this journal.

## Consent

Written informed consent was obtained from the patient for publication of this Case report and any accompanying images. A copy of the written consent is available for review by the Editor-in-Chief of this journal.

## Authors’ contributions

DF: Methodology, validation, investigation, data curation, writing original draft, writing reviewing and editing, visualization. PC: writing original draft, methodology, investigation. MB: writing reviewing and editing, visualization. DR: writing, reviewing and editing. RDH: writing, reviewing and editing. JRT: writing, reviewing and editing. MHR: conceptualization, methodology, writing reviewing and editing. All authors read and approved the final manuscript.

## Registration of research studies

N/A.

## Guarantor

Dr’s David Forner and Matthew Rigby are to be considered the co-guarantor’s for this manuscript.

## Provenance and peer review

Not commissioned, externally peer reviewed.
